# The Weariness of Dental Practice

**Published:** 1891-04

**Authors:** 


### The Weariness of Dental Practice.

It occurs to me that I have not read in the journals devoted to dentistry any thing about the patient who spits everywhere rather than in the spittoon provided at his side. Especially is this the case when you are operating for the removal of calcic deposits. Although the incrustations may have been there for a quarter of a century, yet when they are loosened he (of course a man(?) is seized with a frantic desire to get the particles out of his mouth, and fires them right and left, or straight ahead, and if he misses your eyes or face he is sure to make it tell on your window, or upon the dainty curtain which your wife or sweetheart has placed before the window. These things make life a burden.

Perhaps my city brother, with his aristocratic patients, may say that none of them do such outlandish things, but let me whisper in his ear, that he probably prevaricates. A twin brother to the man who wont spit in the spittoon is the man who sits down in your operating chair with a cigar in his mouth, and when he removes it places it on your operating table so as to be within easy reach during pauses of work. If my city brother dosen't prevaricate when he says he hasen't any spit-on-the-window-patients, he surely lies if he denies having any of this latter class, for they are found always in the "400." They are swell society men with a touch of cockney English, just the fellows who place the dental operating room upon the same plane as that of the hairdresser. Oh! how it does my heart good to crush these pigs. If *every* dentist would do the same thing there would be less of this particular "weariness." There is another patient somewhat known here in the country, and I am sure my city brethren will recognize him at once. He is the man who breaks an engagement just at the most important date, when the tooth had after long care become ready to fill, and when it becomes bad again will drop in upon you at all outlandish times and insist upon having attention. You will see him just as your regular patient is about taking the chair, and he will beg for "just a second," or, if he don't come then he will catch you after you have your overcoat on and are starting for lunch. If he meets you at the foot of the stairway or elevator it will be all the same, the old story of "how I have suffered," and you must go back and look at it. If he don't catch you at noontime then he will corral you sure about lamplighting, when after a hard day's work your are preparing to go from your office. This is a never-ending "weariness." The way to beat this class is to bring them up with a sharp turn. Give them a second trial, and if they fail again, then tell them to go elsewhere. You can not convert one of these break-appointments-drop-in-on-you patients if the disease gets well commenced. "They are joined to their idols, let them alone."

Another weariness often commented upon is the patient who arrives when your office is full of those waiting, and loudly declares that "the filling you put in for me has come out," and upon examination you find your filling in good shape but a large cavity in an adjoining tooth, or very likely in another part of the mouth entirely, from that upon which you have formerly operated, and which is evidently new and as unacquainted with fillings as a new-born babe is of clothes. She will say (for this time it is generally a woman) "why, is that so? I thought you filled it." But of course she never thinks of the injury she has done your reputation by her proclamation to your waiting patients, or perhaps to all her acquaintances by saying that Dr. So-and-so filled her teeth and the fillings have all come out," nor does she undo the injury by going to them and correcting the statement. This class of patients seem to think that when they once have their teeth put in shape that no further decay can appear in the mouth, and if it does, it must be one already filled. This is a "weariness" which is enough to drive a man to drink, and it is universal. The best way in this case is to drop every thing, seat this patient in the reception room among the persons who have heard her statement, and prove to her at once that she is wrong. Be sure that every body in the room hears what you say.

Now, Mr. Editor, you may publish this if you want to, or trim it up to suit your taste or space. I feel relieved already to know that I have had my say upon a few of the things that make up "The Weariness of Dental Practice." A COUNTRY DENTIST, in *Western Dental Journal.*

